# 4D trajectory prediction for inbound flights

**DOI:** 10.3389/fnbot.2025.1625074

**Published:** 2025-09-17

**Authors:** Weizhen Tang, Jie Dai

**Affiliations:** ^1^Civil Aviation Ombudsman Training College, Civil Aviation Flight University of China, Guanghan, China; ^2^College of Air Traffic Management, Civil Aviation Flight University of China, Guanghan, China

**Keywords:** 4D trajectory prediction, multi-step prediction, DBO algorithm, RCBAM network, modal decomposition

## Abstract

**Introduction:**

To address the challenges of cumulative errors, insufficient modeling of complex spatiotemporal features, and limitations in computational efficiency and generalization ability in 4D trajectory prediction, this paper proposes a high-precision, robust prediction method.

**Methods:**

A hybrid model SVMD-DBO-RCBAM is constructed, integrating sequential variational modal decomposition (SVMD), the dung beetle optimization algorithm (DBO), and the ResNet-CBAM network. Innovations include frequency-domain feature decoupling, dynamic parameter optimization, and enhanced spatio-temporal feature focusing.

**Results:**

Experiments show that the model achieves a low longitude MAE of 0.0377 in single-step prediction, a 38.5% reduction compared to the baseline model; in multi-step prediction, the longitude R2 reaches 0.9844, with a 72.9% reduction in cumulative error rate and an IQR of prediction errors less than 10% of traditional models, demonstrating high accuracy and stability.

**Discussion:**

Experiments show that the model achieves a low longitude MAE of 0.0377 in single-step prediction, a 38.5% reduction compared to the baseline model; in multi-step prediction, the longitude R2 reaches 0.9844, with a 72.9% reduction in cumulative error rate and an IQR of prediction errors less than 10% of traditional models, demonstrating high accuracy and stability.

## 1 Introduction

In recent years, the global aviation industry has experienced rapid growth, with air traffic volumes continuing to rise. According to forecasts and observational data, flight density at major airspace nodes and busy airports has significantly increased, directly leading to frequent flight delays, growing airspace resource constraints, and increased workload for air traffic controllers, among other serious issues (Ma et al., 2024). To effectively address the growing pressure on air traffic control (ATC) systems caused by increasing air traffic volume and to enhance airspace operational efficiency and safety, the International Civil Aviation Organization (ICAO) has established TBO as the core strategy for future global air traffic management (ATM) (Ramasamy et al., 2014). The core of TBO lies in utilizing high-precision four-dimensional data throughout an aircraft’s entire flight cycle to optimize flight paths and improve airspace utilization efficiency (Zeng et al., 2022). TBO as the core strategy for future global ATM (Ramasamy et al., 2014). The core of TBO lies in utilizing high-precision four-dimensional trajectory information throughout the entire flight cycle of an aircraft to achieve real-time information sharing and dynamic collaborative decision-making among multiple stakeholders, including air traffic control, airports, airlines, and aircraft (Zeng et al., 2022; Hao et al., 2018). This transformation imposes unprecedentedly high demands on the accuracy and reliability of trajectory prediction. The Civil Aviation Administration of China (CAAC) Air Traffic Management Bureau explicitly listed TBO and 4D trajectory prediction as key pillars for achieving ATM modernization in the “Implementation Roadmap for the Civil Aviation Air Traffic Management Modernization Strategy (CAAMS)” published in March 2020 (International Civil Aviation Organization, 2018; Cheng et al., 2020). High-precision, reliable flight path prediction, particularly multi-step prediction capable of anticipating future flight states over an extended period, has become a foundational technology for enhancing air traffic management system operational safety, optimizing airspace resource utilization, reducing flight delays, lowering control workload, and ultimately realizing the TBO vision. Compared to single-step prediction, which only forecasts the state at the next moment, multi-step prediction provides a longer forecast horizon. This is of decisive significance for real-time dynamic adjustment of flight paths, early detection and resolution of potential flight conflicts, and optimization of traffic management, making it a critical factor in ensuring flight safety and efficiency in high-density airspace.

Currently, methods regarding short-term trajectory prediction can be broadly categorized into three types of methods: mass motion-based, state estimation-based, and machine learning-based. Trajectory prediction based on mass motion (Peng et al., 2005) is to consider the air vehicle as a particle, analyze the force on it, and establish a prediction model by combining the kinematic model and the aircraft parameters. This method has the problem of requiring too many parameters related to the aircraft itself and the kinematics. Trajectory prediction based on state estimation (Tang et al., 2020) regards the motion process of an airplane as a state transfer process, constructs a state transfer matrix through the equations of motion, and investigates the relationship between the position at a future point in time and the states of position, velocity, acceleration, etc. at a historical point in time. However, the model constructed by this method has obvious limitations in dealing with the trajectory data, which is difficult to cope with the complex nonlinear relationships and external disturbances in it, and the computational complexity is high.

Machine learning has been increasingly applied to trajectory prediction by mining hidden information in large-scale data, constructing neural networks, and capturing nonlinear relationships, which is essential for improving prediction accuracy. Short-term trajectory prediction can be divided into single-step and multi-step prediction. Classical neural networks such as BP, LSTM, GRU, and their RNN variants have been widely used; however, their recursive mechanisms limit computational efficiency and parallelization. To address these issues, convolutional neural networks (CNN) and temporal convolutional networks (TCN) have been introduced into trajectory prediction. In multi-step prediction, Jiao and Yang (2024) combined TCN with Multi-Scale Convolution and spatio-temporal dual attention to improve accuracy and continuity. Encoder–decoder structures have also been adopted to capture long-term dependencies. For example, Lu et al. (2024) proposed a Seq2Seq model integrating attention and exponential decay sampling, while Huang and Ding (2022) developed a TCN–BiGRU encoder–decoder optimized by Bayesian algorithms. Shi et al. (2024) further enhanced efficiency with a GRU–TCN Seq2Seq model using temporal pattern attention. Hybrid models have also shown promise. Shafienya and Regan (2022) combined CNN–GRU and 3D-CNN with Monte Carlo dropout, reducing prediction error significantly. Chuan et al. (2023) fused convolutional recurrent networks with LSTM to capture navigation attributes and suppress error accumulation. Dong et al. (2023) introduced a TCN–Informer model achieving superior performance across multiple metrics. Other works explored clustering and generative adversarial networks: Zhang and Liu (2025) used K-medoids with CTGAN for mid-to-long-term prediction, while Wu et al. (2022) compared Conv1D-GAN, Conv2D-GAN, and LSTM-GAN, finding Conv1D-GAN most effective. LSTM remains widely applied, as in Liu and Hansen (2018) with an encoder–decoder LSTM and Zhao et al. (2019) with a deep LSTM for robust prediction in complex flight environments.

In the latest research, optimization algorithms are widely used. Li et al. (2025) enhanced the sparrow search algorithm using sine chaotic mapping to optimize the BP (back propagation) neural network. Deng et al. (2024) proposed a novel Quantum Differential Evolution Algorithm with a quantum adaptive mutation strategy and a population state evaluation framework, namely PSEQADE. The results show that PSEQADE exhibits excellent convergence performance, high convergence accuracy, and remarkable stability in solving high-dimensional complex problems. Zhu et al. (2024) proposed a hybrid multi-strategy genetic algorithm (RPIP-GA) based on opposition-based learning, interval probability mutation, and engulfment mechanism to address the airport gate assignment problem. The algorithm effectively improves the performance of solving high-dimensional complex problems. Huang et al. (2024) proposed a novel competitive group optimizer (DMCACSO) that solves large-scale optimization problems (LSOP) through a dynamic multi-competition mechanism and convergence accelerator. Experimental results show that DMCACSO has competitive optimization performance when solving large-scale benchmark functions and performs well in actual feature selection problems. Song and Song (2025) proposed a new adaptive evolutionary multi-task optimization algorithm, MGAD, which significantly improves the performance of multi-task optimization through a dynamic knowledge transfer probability strategy, an improved source task selection mechanism, and an anomaly detection knowledge transfer strategy. Experimental results demonstrate that it is highly competitive in terms of convergence speed and optimization capabilities.

However, existing trajectory prediction research faces three main challenges: (1) limited ability to model complex relationships among multi-dimensional features (e.g., longitude, latitude, altitude, speed); (2) difficulty capturing long-term dependencies in ultra-long sequences with traditional architectures (e.g., CNN, RNN), leading to information loss; and (3) error accumulation in multi-step prediction, reducing long-term accuracy. To address these, this paper proposes the SVMD-DBO-RCBAM hybrid model, which integrates signal decomposition, intelligent optimization, and attention mechanisms. First, SVMD separates noise from valid trajectory signals, producing multi-scale, high signal-to-noise submodes for cleaner and more informative inputs. Second, DBO dynamically optimizes RCBAM hyperparameters via a global–local search, reducing manual tuning and suppressing bias accumulation. Finally, the RCBAM network combines ResNet’s deep feature extraction with CBAM’s dual-channel-spatial attention: channel attention weights feature dimensions (e.g., longitude, latitude, altitude), while spatial attention focuses on critical time windows, improving multi-factor feature fusion and long-term dependency modeling. Overall, the architecture enables fine-grained modeling and dynamic optimization, overcoming limitations of existing methods.

The rest of the paper is organized as follows. Section 2 describes the data sources and preprocessing. Section 3 describes SVMD, ResNet network, CBAM network, DBO, basics. Section 4 describes the construction of the 4D trajectory model and its steps. Section 5 compares the performance of the main model through ablation experiments and analyzes it in comparison with other mainstream models. Finally, Section 6 summarizes and outlooks the results.

## 2 Data collection and pre-processing

### 2.1 ADS-B data

ADS-B historical trajectory data is the data basis for the 4D trajectory prediction in this paper. ADS-B is an aircraft operation monitoring technology. The transmitter of the aircraft onboard equipment sends aircraft information to the ADS-B ground station or other airplanes loaded with ADS-B onboard equipment at a certain period. The specific content includes: sampling time, position, altitude, speed, flight number, heading, climb or descent rate, etc. The experimental data in this paper originates from Flight Sense Technology Company^1^, and some data examples are shown in Table 1. As seen in Table 1: there are problems such as unequal sampling time intervals between neighboring trajectory points, and duplication of latitude and longitude data in different sampling points in the data, which need to be preprocessed.

**Table 1:** Examples of ADS-B raw data.

Sampling time	Altitude/ft	Speed/kt	Direction/°	Latitude/°	Longitude/°
2022-11-07 t08:46:50	8,250	165	217	25.0771	102.9203
2022-11-07 t08:47:05	8,650	167	217	25.0706	102.9143
2022-11-07 t08:47:20	9,050	166	217	25.0641	102.9088
2022-11-07 t08:47:38	9,575	180	216	25.0559	102.9019
2022-11-07 t08:47:50	10,000	194	218	25.0679	102.8949
2022-11-07 t08:48:07	10,400	216	207	25.0378	102.8949
2022-11-07 t08:48:22	10,700	234	203	25.0263	102.8759

### 2.2 Data pre-processing

The preprocessing process includes steps such as outlier processing, track point interpolation, and normalization of the raw data, along with data alignment and data construction.

#### 2.2.1 Handling of outliers

Outlier handling includes 3 cases: missing data, data duplication and abnormal data values. For missing data and data duplication, they can be directly deleted or de-duplicated. For the case of abnormal data values, if the abnormal data is less, the trajectory smoothing algorithm can be used to replace it; if the abnormal data is too much, the track can be deleted directly.

#### 2.2.2 Trajectory point interpolation and data alignment

**Figure 1:**
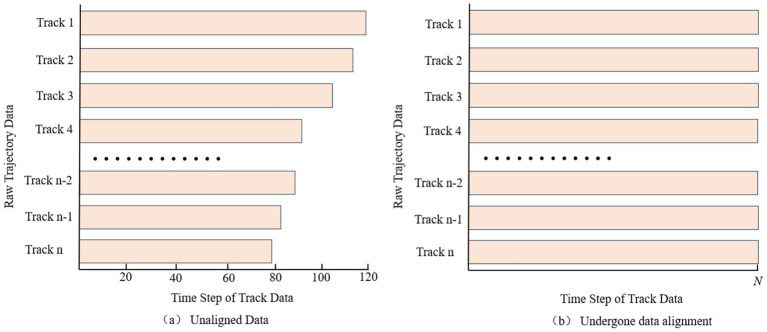
Data alignment pre-processing comparison example chart. Comparison of data alignment pre-processing for trajectory data. **(a)** Unaligned data. **(b)** Undergone data alignment.

In order to solve the problem of different time intervals in historical track data, this paper adopts the cubic spline interpolation method to reconstruct the track features, and sets the same acquisition frequency for each track, so that each track point has the same time interval between them. The interpolated trajectory data have the same time interval, but due to the unequal total time spent on each trajectory, which results in a different number of trajectory points, i.e., the number of time steps is not the same in each trajectory data, see Figure 1a. In order to better learn the overall trend of the trajectory, this paper uniformly samples N (number of time steps) trajectory points for each interpolated whole trajectory, thus realizing the data alignment and meeting the input requirement of uniform number of steps for the trajectory prediction model, see Figure 1b.

#### 2.2.3 Normalization

The trajectory data consists of different feature sequences, and the features have different scales and large differences in data ranges, which may cause the model to be more sensitive to some features and less sensitive to other features during training, making it difficult for the training process to converge. Therefore, in this section, in order to avoid this situation from affecting the prediction results, the trajectory data need to be normalized. By using normalization, the training process can be stabilized, the convergence speed of the model can be improved, the occurrence probability of gradient explosion and gradient disappearance can be reduced, and the generalization ability and prediction accuracy of the model can be improved. This model uses min-max normalization in training to map all values between (0, 1), and the normalization formula is as follows Equation (1):


(1) $$\text{Normalized}(x)=\frac{x-x_{\min}}{x_{\max}-x_{\min}}$$

where Normalized is the normalization result, *x* is the input value of the independent variable, and *max* and *min* are the maximum and minimum values of the corresponding input tensor, respectively.

#### 2.2.4 Data construction

**Figure 2:**
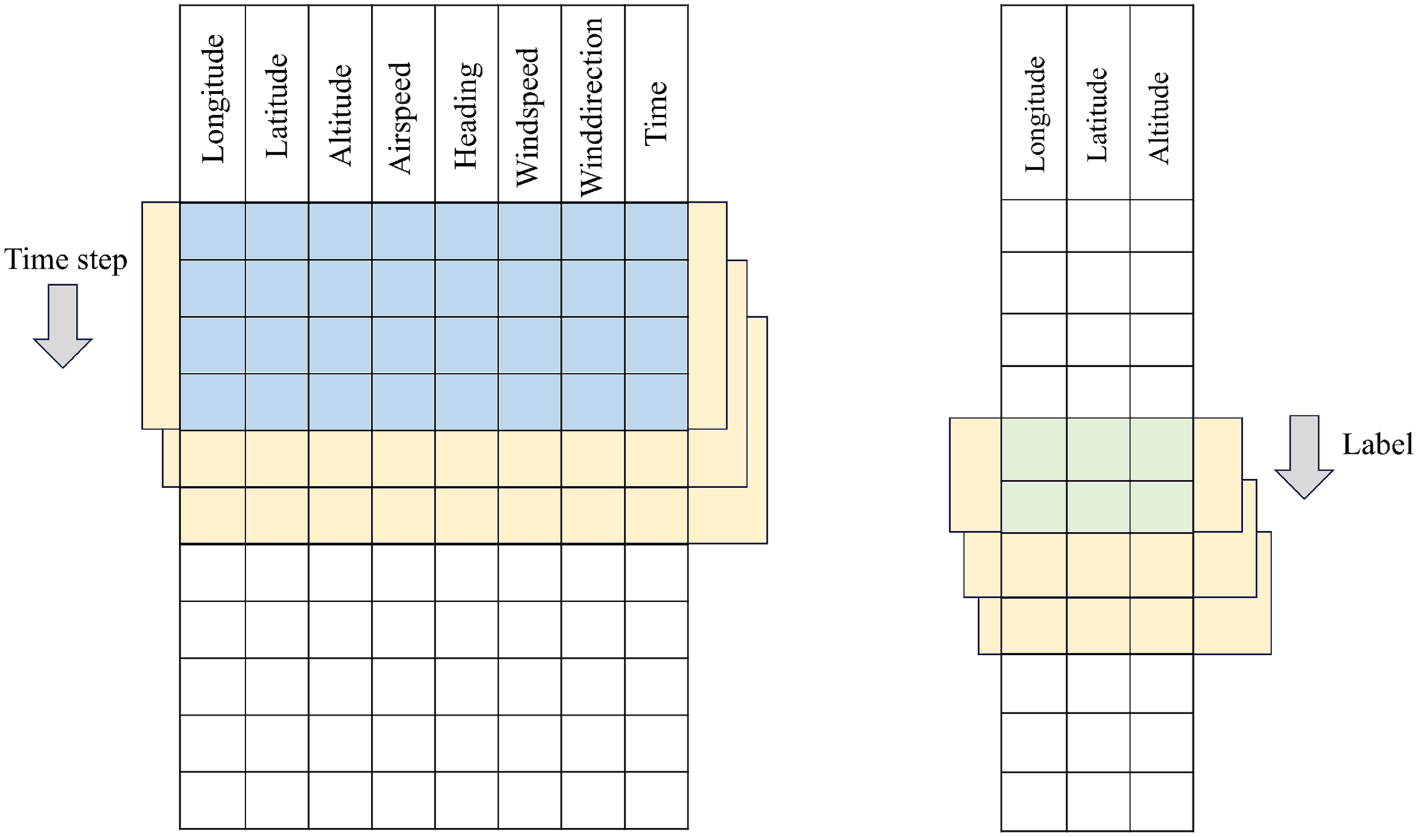
Schematic diagram of single-step time series construction.

**Figure 3:**
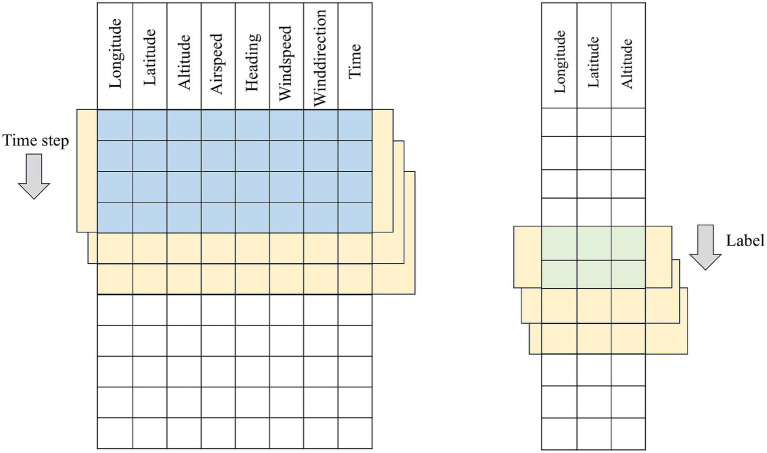
Schematic diagram of multi-step time series construction.

In this section, a collection of historical flight trajectory data of flights = {D1,…, Dm} is constructed and processed, each data contains Features individual features. The data construction adopts the sliding window method, starting from the first row of the trajectory data, the trajectory points of the rows corresponding to the time_step (time window) are selected in chronological order as the input data of the model, and one or more rows of the prediction targets are selected as the label data. Subsequently, the window is moved to the next window of trajectory points to generate the next sample. This process is repeated until the window covers the entire trajectory sequence, and the data construction principle is shown in the construction schematic diagrams of Figure 2 for single-step prediction and Figure 3 for multi-step prediction. The number of samples batch_size individual window combinations are obtained through data construction, as the model training to provide the required input data and labeled data, i.e., training data, validation data, and test data, to construct the 3D tensor of (batch_size, time_step, Features) dataset.

## 3 Basic algorithmic principles

### 3.1 Successive variable modal decomposition (SVMD) algorithm

SVMD is a highly robust signal decomposition method, which is able to decompose a non-smooth signal into a number of modal components with different frequency characteristics step by step without the need to preset the number of modes. The basic idea of SVMD is to extract the intrinsic mode function (IMF) of the signal one by one through an optimization process and retain the residual terms to ensure the reconstruction integrity. The basic idea is to extract the intrinsic modal functions (IMFs) in the signal one by one through an optimization process and retain the residual terms to ensure the reconstruction integrity. The basic expression of SVMD is Equation (2):


(2) $$x(t)=\sum_{l=1}^{L}u_{l}(t)+r_{L}(t)$$

Where x(t) is the original input signal, $u_{l}(t)\;$ denotes the *l-*th order modal component extracted, *L* is the total number of modes extracted, and $r_{L}(t)$ is the residual signal, which contains the portion of the signal that is not explained by all the current modal components.

In order to extract modes with good frequency domain characteristics and mutual independence, SVMD constructs a series of constrained objectives. First, the following optimization objective is constructed to ensure the frequency concentration of each mode Equation (3):


(3) $$\min_{u_{l},w_{l}}\sum_{l=1}^{L}{\left\|\partial_{t}[\delta (t)+\frac{j}{\mathrm{\pi t}}*\mu_{l}(t)]e^{-{\mathrm{jw}}_{l}t}\right\|}^{2}$$

In this equation, $\mu_{l}(t)\;$ denotes the *l*-th modal component, $w_{l}$ is its corresponding center frequency, δ(t) is the unit impulse function, *j* denotes the imaginary unit, * denotes the convolution operation, $\partial_{t}$ is the derivative operation with respect to time, which denotes the rate of change of the frequency feature, and the exponential term $e^{-{\mathrm{jw}}_{l}t}$ is used to move the modes to the baseband. The overall goal of the equation is to minimize the derivative energy of the FM signal so that the modes have minimum bandwidth in the frequency domain.

In order to achieve frequency separability between modes, a band-pass filter is introduced with a frequency response defined as Equation (4):


(4) $$H_{l}(f)=\frac{1}{1+{\left(\frac{f-w_{l}}{\sigma }\right)}^{2n}}$$

In this expression, $H_{l}(f)$ is the frequency response function of the filter used for the *l*-th mode, *f* is the frequency variable, $w_{l}$ is the center frequency of the mode, σ is the scale factor that regulates the bandwidth of the filter, and *n* is the order of the filter, which is used to control the steepness of the filter response. With this filter, components in the frequency domain close to $w_{l}$ can be extracted from the original signal to construct the corresponding time domain modal components Equation (5):


(5) $$u_{l}(t)=h_{l}(t)*x(t)$$

Where $h_{l}(t)$ is the time-domain impulse response corresponding to $H_{l}(f)$ , x(t) is the original signal, and ∗ denotes the convolution operation. This expression describes the process of extracting the *l*-th mode from the original signal through the filter.

Since there may still be some overlap between the modes, to further improve the independence between the modes, the SVMD introduces another set of optimized filters with a frequency response of Equation (6):


(6) $$\tilde{H_{l}}(f)=\frac{1}{1+{\left(\frac{f-w_{l}}{\rho }\right)}^{2m}}$$

Where $\tilde{H_{l}}(f)$ is the frequency response function of the improved filter, ρ is the new bandwidth control factor for adjusting the frequency band coverage, and m is the order of the filter for adjusting the shape of the frequency response and suppressing the inter-modal spectral interference. Its corresponding time domain filtering relation is Equation (7):


(7) $$u_{l}(t)=\tilde{h_{l}}(t)*x(t)$$

Where $\tilde{h_{l}}(t)$ is the time-domain impulse response function of the filter $\tilde{H_{l}}(f)$ , and other symbols have the same meaning as before.

In addition, to ensure that all extracted modal components and residuals can completely reconstruct the original signal, the following reconstruction constraints need to be satisfied Equation (8):


(8) $$x(t)=\sum_{l=1}^{L}u_{l}(t)+r_{L}(t)$$

Ultimately, the SVMD solution problem is transformed into a weighted minimization problem that combines multiple objectives of the form Equation (9):


(9) $$\min_{u_{l},w_{l}}\{\alpha \cdot J_{1}+\beta \cdot J_{2}+\gamma \cdot J_{3}\}$$

Among them, $J_{1}$ is the frequency concentration objective function of the modes, $J_{2}$ and $J_{3}$ are used to portray the frequency separateness and independence of the modes, respectively, and α , β , γ are hyperparameters used to adjust the importance weights of the three to ensure that the modes meet the physical significance and have good mathematical properties at the same time.

### 3.2 Dung beetle optimization algorithm (DBO)

Dung Beetle Optimizer (DBO) (Xue and Shen, 2023) is an intelligent optimization algorithm that simulates the natural behaviors of dung beetles, featuring high population diversity and excellent search capability. DBO constructs a search mechanism that combines global and local search by simulating the behaviors of pushing balls, dancing, laying eggs, foraging and stealing. The algorithm divides the population into different behavioral roles, and each type of dung beetle has a unique update strategy.

In the push-ball behavior, the dung beetle moves in a straight line along the direction of sunlight with the following position update expression:


(10) $$X_{i}^{t+1}=X_{i}^{t}+k\cdot \mathrm{sgn}(\mathrm{rand}-0.5)\cdot b\cdot e^{-\mathrm{\lambda t}}\cdot (X_{i}^{t}-X_{\text{worst}})$$

Where $X_{i}^{t}$ denotes the position vector of the ith dung beetle in the *t*-th iteration, and $X_{\text{worst}}$ is the position of the least adapted individual in the current population; *k*∈(0, 0.2] is the directional offset coefficient, which is used to control the amplitude of the movement perturbation; *b*∈(0, 1) is the adjustment parameter of the movement step size; *λ* is the coefficient of the sunlight intensity decay over time; *rand*∈(0, 1) is the uniformly distributed random number; and *sgn* is a symbolic function, whose value is 1 or −1, used to randomly control the offset direction.

When the dung beetle encounters an obstacle during its movement, its behavioral pattern changes to “dancing,” exploring new paths by changing the direction of movement, and its position update expression is:


(11) $$X_{i}^{t+1}=X_{i}^{t}+\theta \cdot \mathrm{rand}$$

Here, *θ* ∈ [0, *π*] denotes the angular magnitude of the dancing direction, which is a random angle from 0 to π, and *rand* ∈ (0, 1) is the random step factor. Whether to perform the dancing behavior is controlled by another random variable $r_{2}$ ∈ (0, 1), when $r_{2}$ exceeds a certain set threshold, the dung beetle will change its direction, otherwise it continues to push the ball in a straight line.

In the spawning behavior, the dung beetle performs a local search around a locally optimal position with the following position update strategy:


(12) $$X_{i}^{t+1}=X^{*}+\mathrm{rand}\cdot (\mathrm{Ub}-\mathrm{Lb})\cdot (1-\frac{t}{T_{\max}})$$

Where $X^{*}$ denotes the location of the locally optimal individual, Ub and Lb are the upper and lower bounds of the problem definition domain, respectively, $T_{\max}$ is the maximum number of iterations, *t* is the current number of iterations, and *rand* ∈ (0, 1) is a random variable used to control the degree of perturbation. The strategy realizes adaptive control with the local search range shrinking over time.

The foraging behavior simulates the dung beetle’s jumping search toward the global optimal solution with the update formula:


(13) $$X_{i}^{t+1}=X_{\text{best}}+\mathrm{rand}\cdot (\mathrm{Ub}-\mathrm{Lb})\cdot (1-\frac{t}{T_{\max}})$$

In this equation, $X_{\text{best}}$ denotes the position of the optimal individual of the population in the current iteration, and the meaning of the rest of the parameters is the same as that in Equation (12). This update strategy ensures that the population searches around the optimal solution with appropriate stochastic perturbations, maintaining the global exploration capability.

Finally, the stealing behavior performs individual position updating by combining the global optimal position with a noise-bearing perturbation, which is expressed as:


(14) $$X_{i}^{t+1}=X_{\text{best}}+S\cdot g$$

where *S* is a step factor to control the magnitude of the perturbation and *g* is a random vector obeying a standard normal distribution to introduce randomness to jump out of the local optimum. This mechanism strengthens the algorithm’s ability to explore in the later stages of convergence.

### 3.3 ResNet network

Residual Network (ResNet) is a deep convolutional neural network architecture that solves the problem of training difficulties as the depth of the network increases, and in particular solves the gradient vanishing and gradient explosion problems (Wei, 2024). The core idea of ResNet is the introduction of Residual Learning (RL). Conventional neural network layers are directly fitted with a mapping relationship between the bottom input x and the top output F(x), i.e., y = F(x). Such a mapping can lead to the introduction of errors, especially if the number of network layers is large and the network is deep. In contrast, ResNet lets each layer of the network learn the residual mapping, i.e., the difference between the input and the output. If the input is x, the residual learning part is F(x), and y is the output, when F(x) learns a residual close to zero, then y is close to x. The basic blocks of ResNet can be expressed as follows:


(15) y=F(x)+x

The basic building blocks of ResNet are residual blocks. Each residual block contains two or three convolutional layers, as well as a skip connection that skips over these layers. This connection is made by simply adding the inputs of the block to its outputs, allowing the gradient of the deep network to also pass directly through these skip connections. During training, the gradient is propagated back through both F(x) and x paths by the backpropagation algorithm. If F(x) learns a residual close to zero, then this residual will have a small effect on the gradient propagation, thus avoiding the gradient vanishing problem. In summary, ResNet, by introducing the residual learning mechanism, makes the network able to maintain convergence even if more levels are added, effectively solves the gradient vanishing and gradient explosion problems in deep neural network training, and thus realizes the construction of deeper network structures and the extraction of deeper features.

### 3.4 CBAM network

**Figure 4:**
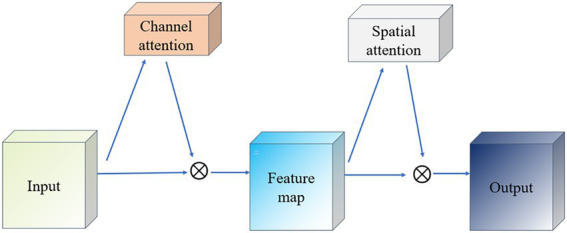
Schematic diagram of CBAM network.

Convolutional Block Attention Module (CBAM) is a lightweight attention mechanism module (Fan et al., 2020), which is designed to enhance the representation of features in convolutional neural networks. CBAM enhances the feature map by sequentially integrating spatial and channel attention mechanisms in a sequential order to adaptively weight the feature map as a way to improve the network’s ability to capture important information. CBAM consists of two main components: Channel Attention and Spatial Attention (Bai et al., 2018), which focus on different feature dimensions. The working principle of CBAM is shown in Figure 4. The input feature maps are first passed through the Channel Attention module, which evaluates the input feature map and assigns different weights to different channels. Next, the feature maps after adjusting the channel weights are passed to the spatial attention module, which further emphasizes spatially important regions. Finally, the channel and spatial attention maps are multiplied with the original feature map to achieve adaptive feature adjustment.

**Figure 5:**
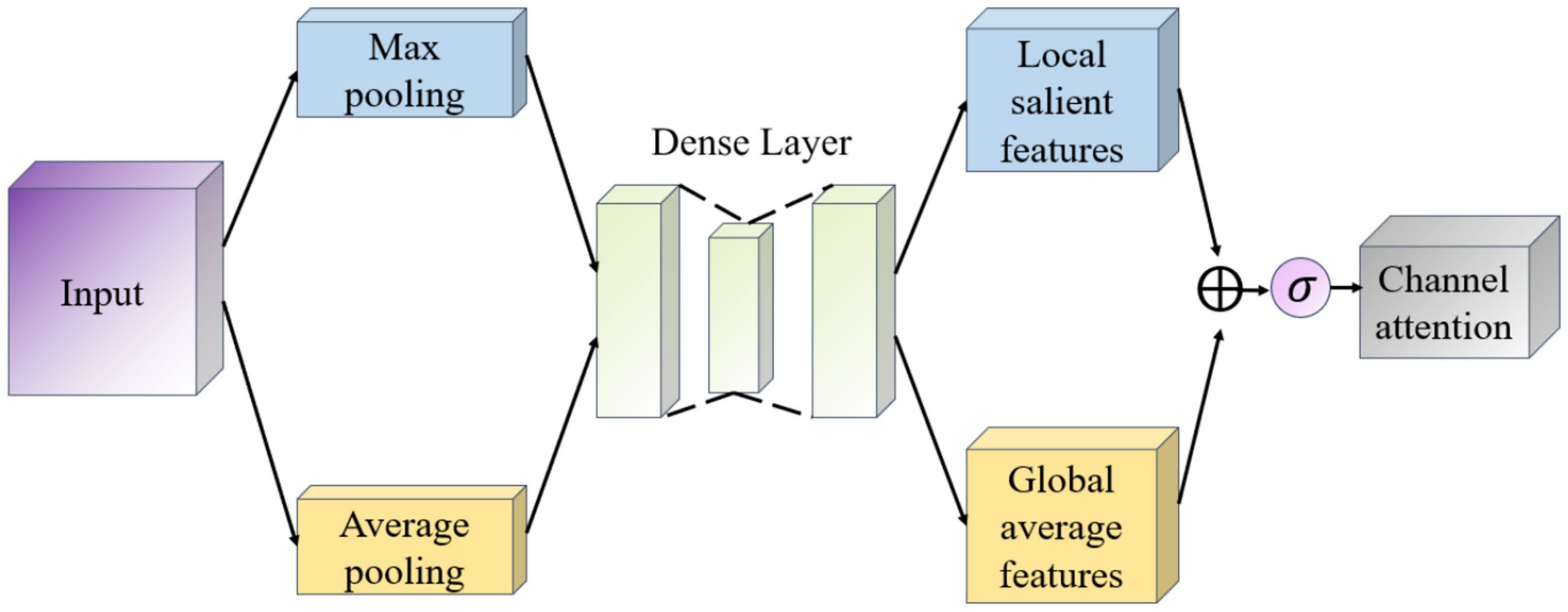
Channel attention module schematic.

The goal of the Channel Attention module (Channel Attention) is to determine the importance of individual channels (i.e., different feature maps) and works as shown in Figure 5. It first uses global average pooling and global maximum pooling operations to generate two different feature maps, which capture the distribution information of the channels, respectively. Then, these two feature maps are fed into a shared fully connected layer, which contains a hidden layer. Finally, the outputs of these two MLPs are summed by elements and a Sigmoid activation function is applied to obtain the attention weights for each channel.

**Figure 6:**
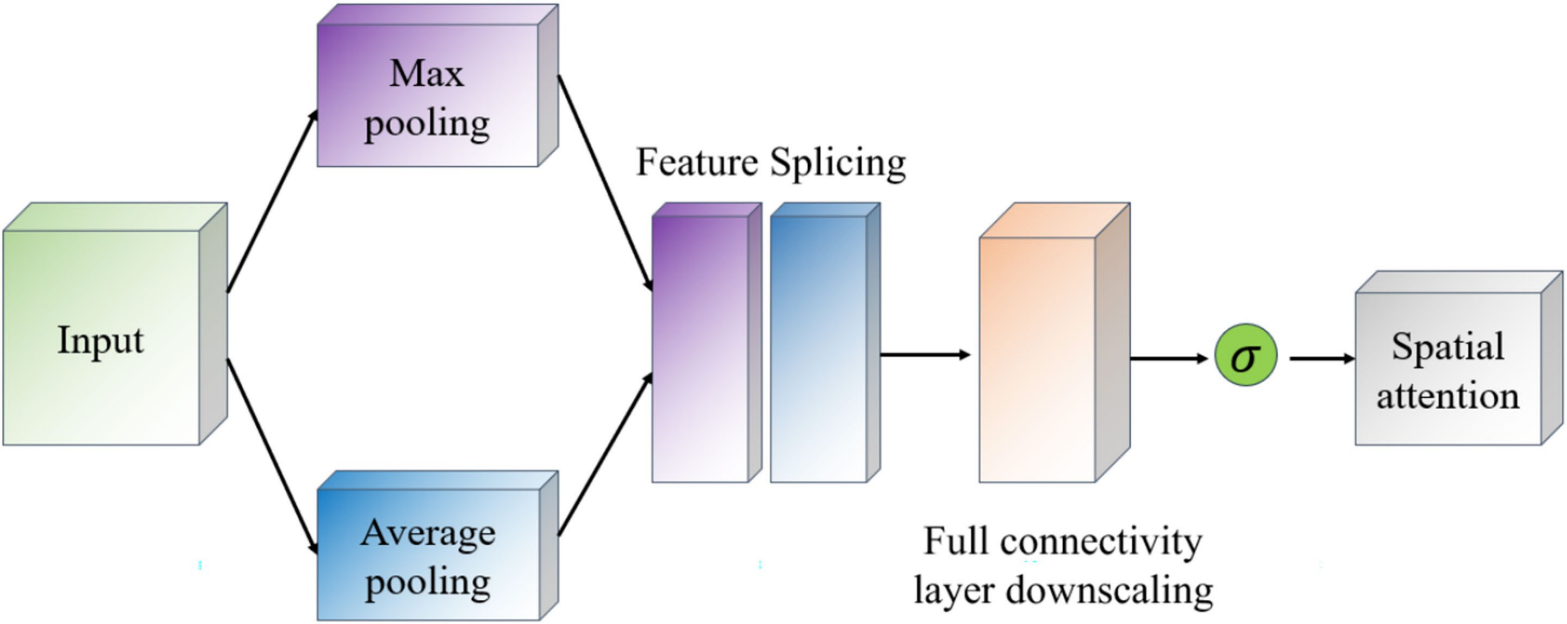
Schematic diagram of the space attention module.

The Spatial Attention module (Spatial Attention) follows Channel Attention and aims to highlight important regions in the spatial dimension and works as shown in Figure 6. This module uses the output of channel attention and processes it to generate a two-dimensional attention map. Specifically, it first performs average pooling and maximum pooling in the channel direction on the input feature map to generate two 2D feature maps, which are then stitched together in the channel dimension and passed through a convolutional layer to produce the final spatial attention map. This attention feature map is also activated by a Sigmoid function in order to weight the original input feature map.

### 3.5 ResNet-CBAM model

**Figure 7:**
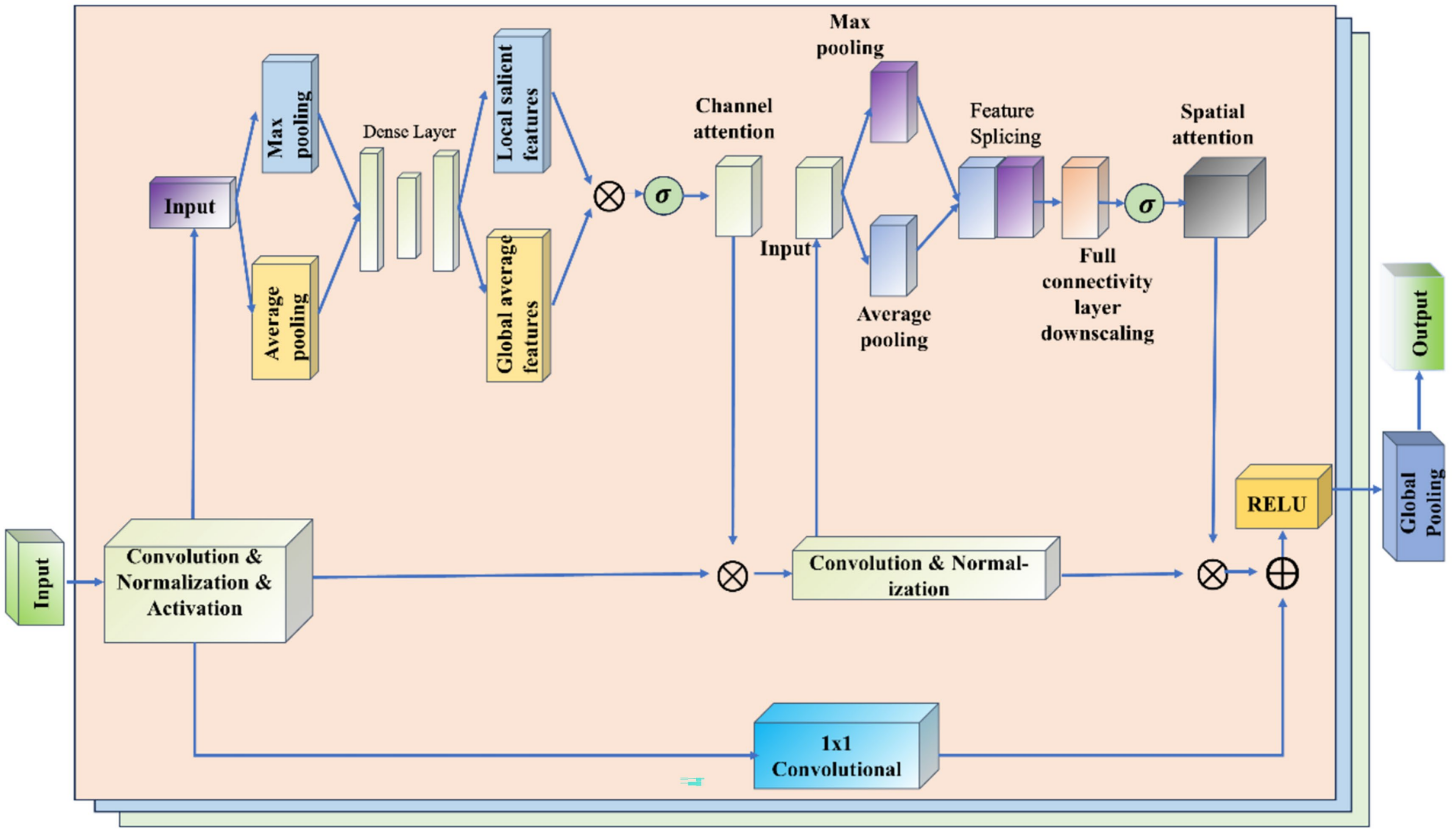
Working schematic of the RCBAM model.

The ResNet-CBAM (RCBAM) model is essentially a combination of a convolutional neural network and an attentional mechanism designed for feature extraction and prediction of trajectory data from a spatial and state change perspective. Figure 7 shows the working schematic of the RCBAM model.

ResNet serves to efficiently extract high-level features from flight track data using deep structure. By treating the sequence data as a one-dimensional image (with time as the width), the change of features over time is captured. Meanwhile residual learning is introduced to solve the problem of overfitting and network degradation that deep learning models are prone to when dealing with complex time series prediction tasks, and to solve the problem of gradient vanishing and gradient explosion in deep networks. The role of CBAM’s channel attention is to allow the model to dynamically assign weights to each input feature, thereby highlighting the features that are most helpful for prediction. The role of CBAM’s spatial attention is to dynamically assign weights to each specific point in time, thereby highlighting the time windows that are most helpful for prediction. The combination of the two CBAM attention mechanisms allows the model to identify both important feature channels and also features at important moments in the trajectory sequence. This helps to construct more refined prediction models that take into account both time dependence and feature importance.

## 4 SVMD-DBO-RCBAM trajectory prediction models

### 4.1 Predictive modeling process

**Figure 8:**
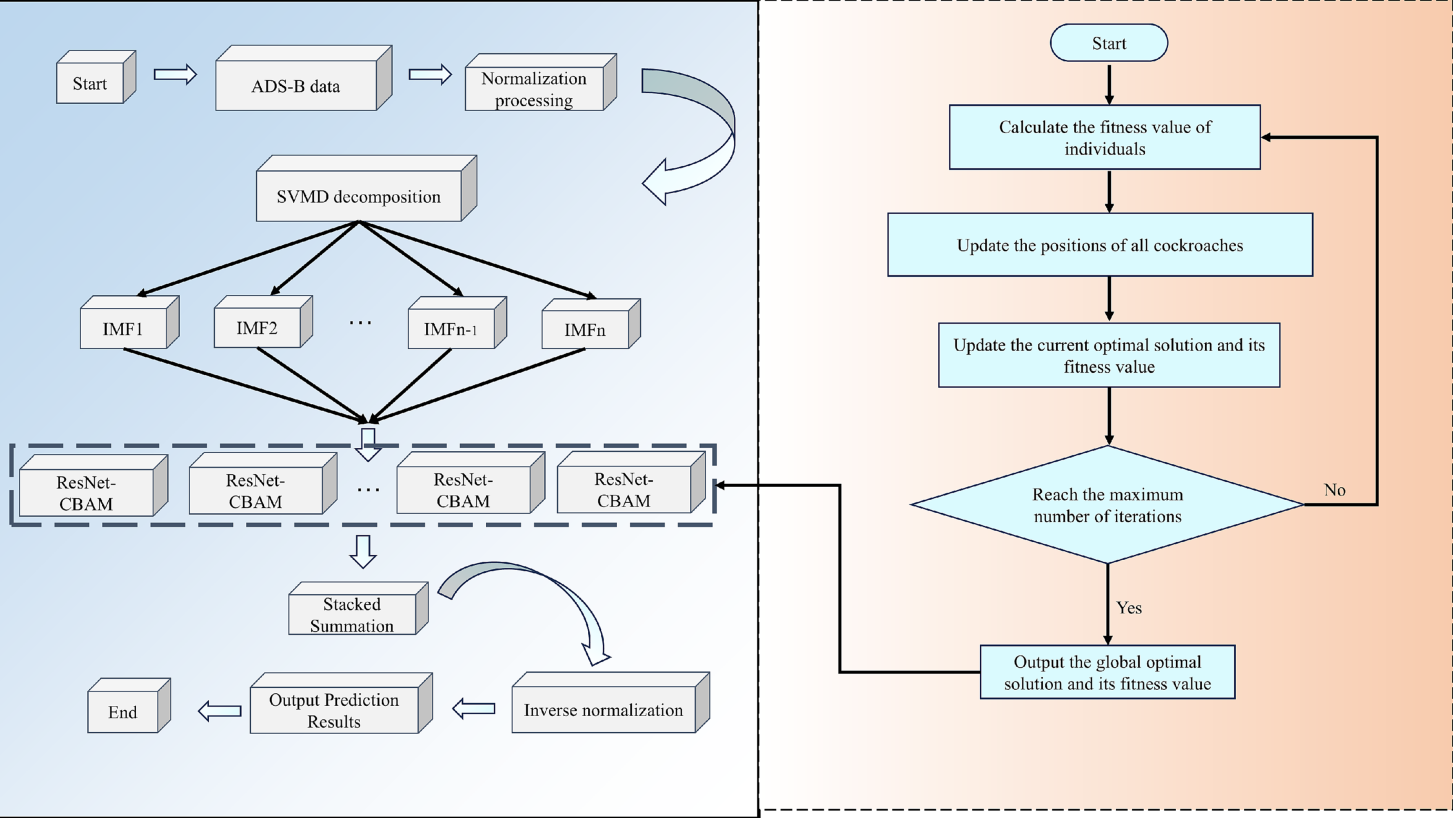
Flowchart of the SVMD-DBO-ResNet-CBAM prediction model.

This paper proposes a hybrid prediction model that integrates SVMD, DBO, ResNet, and CBAM to achieve high-precision prediction of aircraft four-dimensional trajectories. The model aims to overcome the limitations of single models in terms of feature extraction capabilities, frequency domain modeling, and parameter tuning, thereby achieving deep integration of multi-scale spatiotemporal information. Its structure is shown in Figure 8, and the specific process is as follows:

1. Data preprocessing

First, the raw trajectory data collected is cleaned to remove data points with missing values, abnormal changes, or noise interference, ensuring the stability and effectiveness of subsequent modeling. This step plays a critical role in ensuring data quality throughout the process.

1. Data partitioning and normalization

The cleaned data is divided into training, validation, and test sets according to a ratio (8:1:1) to avoid overfitting and enhance the model’s generalization ability. Additionally, the minimum-maximum normalization method is applied to uniformly map all features to the [0, 1] interval, which not only improves numerical stability but also accelerates the convergence speed of the neural network model.

1. Modal decomposition (SVMD)

The SVMD algorithm is used to decompose multi-dimensional track sequences, breaking down the original non-stationary time series into several intrinsic modal functions (IMFs) with different frequency characteristics. Compared to traditional EMD or VMD, SVMD has stronger adaptability and robustness, does not require pre-specifying the number of modes, and can more accurately extract time-frequency features from track data, effectively reducing interference from complex dynamic backgrounds.

1. DBO algorithm initialization

During the parameter optimization phase, the DBO algorithm, which incorporates biological behavior simulation features, is used to replace traditional grid search or random search methods. The initialization phase includes generating the positions of population individuals, setting the fitness function, and dividing behavioral roles (pushing balls, laying eggs, foraging, stealing), among others. This design introduces multiple behavioral patterns, enhancing global search capabilities and preventing getting stuck in local optima.

1. Model parameter optimization and iteration (DBO)

DBO is used to automatically optimize the key hyperparameters of the ResNet-CBAM model, including the number of convolutional layer filters, batch size, learning rate, and number of epochs. In each iteration, DBO updates the positions of individuals, feeds back their fitness based on the model’s performance on the training and validation sets, dynamically guides the optimization path, improves prediction performance, and reduces the cost of manual parameter tuning.

1. Feature extraction and prediction (ResNet-CBAM)

Each IMF subcomponent obtained from SVMD decomposition is input into the optimized ResNet-CBAM model. The ResNet module effectively addresses the gradient vanishing and degradation issues in deep network training through residual connection mechanisms, enhancing feature expression depth. The CBAM module further applies channel attention and spatial attention to the extracted feature maps, automatically focusing on critical time windows and important variable channels, thereby improving sensitivity and discriminative power toward abnormal trajectory changes. Finally, the prediction results of each subcomponent are stacked and summed to comprehensively construct the final flight path prediction value.

1. Denormalization and result output

The normalized prediction results are denormalized to restore them to their original physical quantity scale, meeting practical application requirements. The final four-dimensional trajectory prediction results combine accuracy, timeliness, and interpretability, providing theoretical and technical support for tasks such as flight safety management, trajectory planning, and anomaly detection.

## 5 Simulation verification and analysis

### 5.1 Experimental data and experimental environment

The trajectory dataset used in this paper is the real ADS-B historical trajectory data of inbound flights at Zhuhai Jinwan Airport, which retains the trajectory features such as time, speed, altitude, longitude, latitude, etc. The dataset is stored in the form of csv. Based on this dataset, a 4D trajectory prediction model based on neural network is constructed for trajectory feature and position prediction. From the whole dataset, about 2,842 complete trajectories, totaling 288,200 trajectory points, were intercepted, screened, and retained. The whole data were divided into training set, validation set and test set according to the ratio of 8: 1: 1.

The experimental equipment is a laboratory desktop with an Intel(R) Core(TM) i7-10700 CPU @ 2.90GHz, 2.90GHz and 16GB of RAM on board.

### 5.2 Indicators for model evaluation

In order to compare the performance of different algorithms, this paper uses the Root mean square error (RMSE), Mean Absolute Error (MAE) and Mean absolute percentage error (MAPE) and the coefficient of determination ( $R^{2}$ ) were used as evaluation indexes. Among them, the smaller the values of MAE, RMSE and MAPE are, the better the model prediction effect is; the closer the value of $R^{2}$ is to 1, the better the fitting effect of the prediction model is. The specific calculation formula is as follows.


(16) $$\text{RMSE}=\sqrt{\frac{1}{n}\sum_{i=1}^{n}{\left(y_{i}-Y_{i}\right)}^{2}}$$

(17) $$\mathrm{MAE}=\frac{1}{n}\sum_{i=1}^{n}|y_{i}-Y_{i}|$$

(18) $$\text{MAPE}=\frac{1}{n}\sum_{i=1}^{n}|\frac{y_{i}-Y_{i}}{y_{i}}|\times 100\%$$

(19) $$R^{2}=1-\frac{\sum_{i=1}^{n}{\left(y_{i}-Y_{i}\right)}^{2}}{\sum_{i=1}^{n}{\left(y_{i}-\bar{y_{i}}\right)}^{2}}$$

where *n* is the number of samples, $y_{i}$ is the actual value of the samples, $Y_{i}$ is the predicted value of the model, and $\bar{y_{i}}$ is the summed average of the actual values of the samples.

### 5.3 Algorithmic optimization of network hyperparameters

Adaptive Optimization of Hyperparameters in ResNet-CBAM Neural Networks Using DBO. The main hyperparameters in the ResNet-CBAM model are learning rate, residual block configuration, number of filters, epoch and batch_ size, and the corresponding range of optimization for each hyperparameter is shown in Table 2.

**Table 2:** Hyperparametric optimization range.

Hyperparameters	Optimization range
Learning rate	[0.001,0.005]
epoch	[50,200]
batch_size	[0,128]
residual block configuration	[2,4]
number of filters	[64,256]

**Figure 9:**
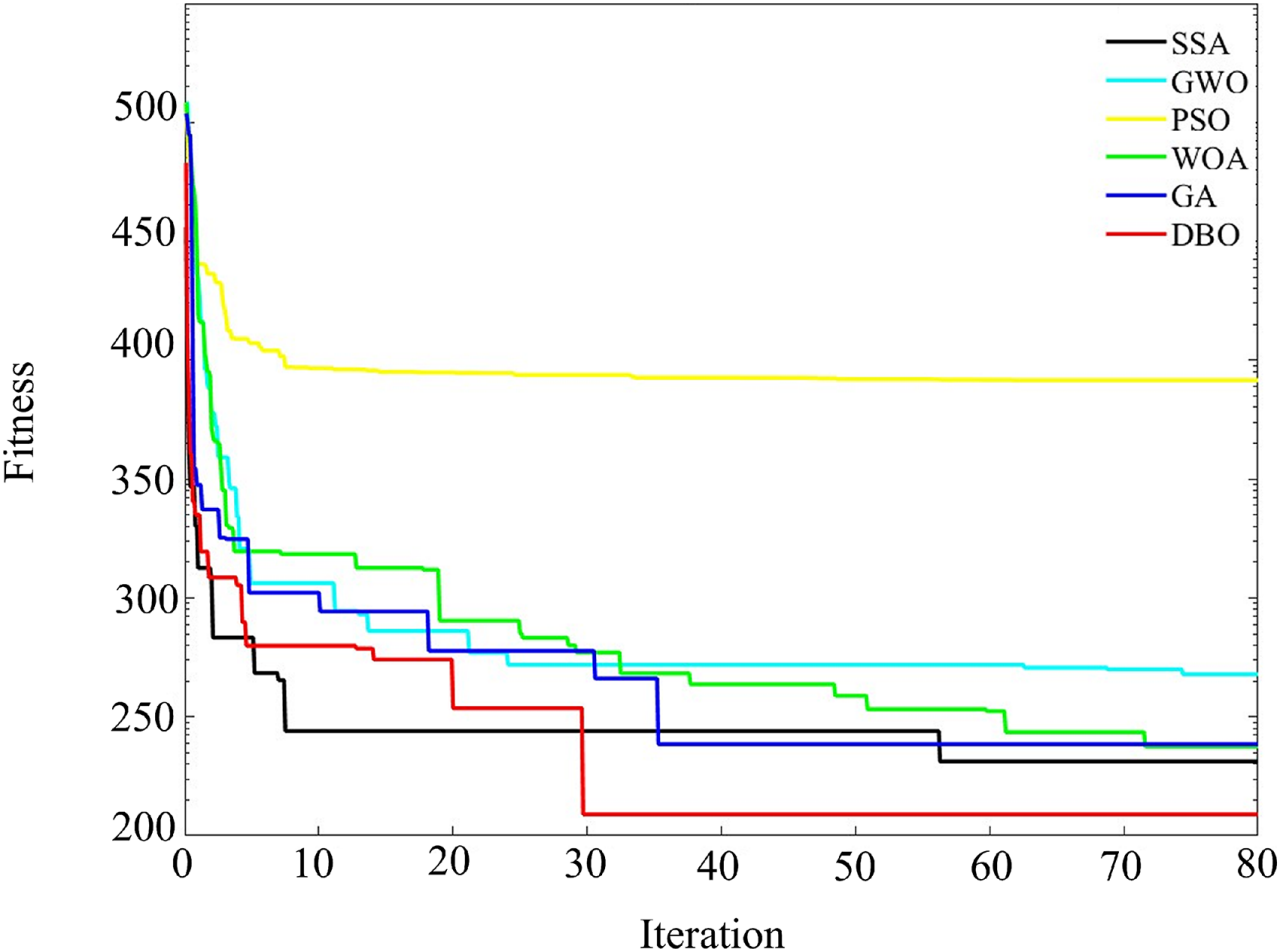
Fitness curve comparison chart.

In order to verify the effectiveness of DBO on ResNet-CBAM hyperparameter optimization, the sparrow search algorithm (SSA), gray wolf optimization algorithm (GWO), particle swarm optimization algorithm (PSO), whale optimization algorithm (WOA), and genetic algorithm (GA) were used to optimize the hyperparameters of ResNet-CBAM, respectively, and the initial population number of each algorithm was set to 100, and the maximum iteration number is set to 80, and the parameter settings of the three algorithms are shown in Table 1. The fitness curve of the optimization process of the optimization of the search parameters is shown in Figure 9, using the mean absolute error (MAE) as the fitness function.

The comparison results of the optimization algorithms in the figure show that the DBO algorithm is significantly better than the other algorithms in terms of convergence speed and final fitness. DBO exhibits rapid convergence speed in the initial iterations and significantly reduces the fitness value, indicating its efficiency in global search and its ability to locate the optimal solution quickly. In contrast, GWO and WOA have higher final adaptation values than DBO due to the lack of local search capability, despite the convergence advantage in the initial iteration. SSA and GA show a smooth decreasing trend, but still fall short of the optimization effect of DBO. Overall, DBO has an excellent ability to balance global and local search, highlighting its ability to quickly locate the optimal solution in the complex problem space.

Encode the five hyperparameters of ResNet-CBAM (learning rate, residual block configuration, number of filters, epoch, batch size) into a vector and randomly initialize multiple candidates within their respective preset ranges. The DBO algorithm simulates the behaviors of dung beetles, such as rolling balls, dancing, laying eggs, foraging, and stealing, to perform both large-scale global exploration and performs local detailed searches within the parameter space. After each iteration, all candidates are evaluated and ranked based on the mean absolute error (MAE) on the validation set. DBO guides “poor-performing” candidates toward more optimal regions while maintaining population diversity. After a predetermined number of iterations, the algorithm automatically selects the vector with the lowest MAE, which is mapped to the final hyperparameter values in our report (learning rate = 0.002, residual block configuration = 2, number of filters = 128, epoch = 75, batch_size = 64). The hyperparameter optimization search results are shown in Table 3.

**Table 3:** Hyperparameter optimization results.

Hyperparameters	Optimization range
Learning rate	0.002
epoch	75
batch_size	64
residual block configuration	2
number of filters	128

### 5.4 Experiments on attention weights of the CBAM module

**Figure 10:**
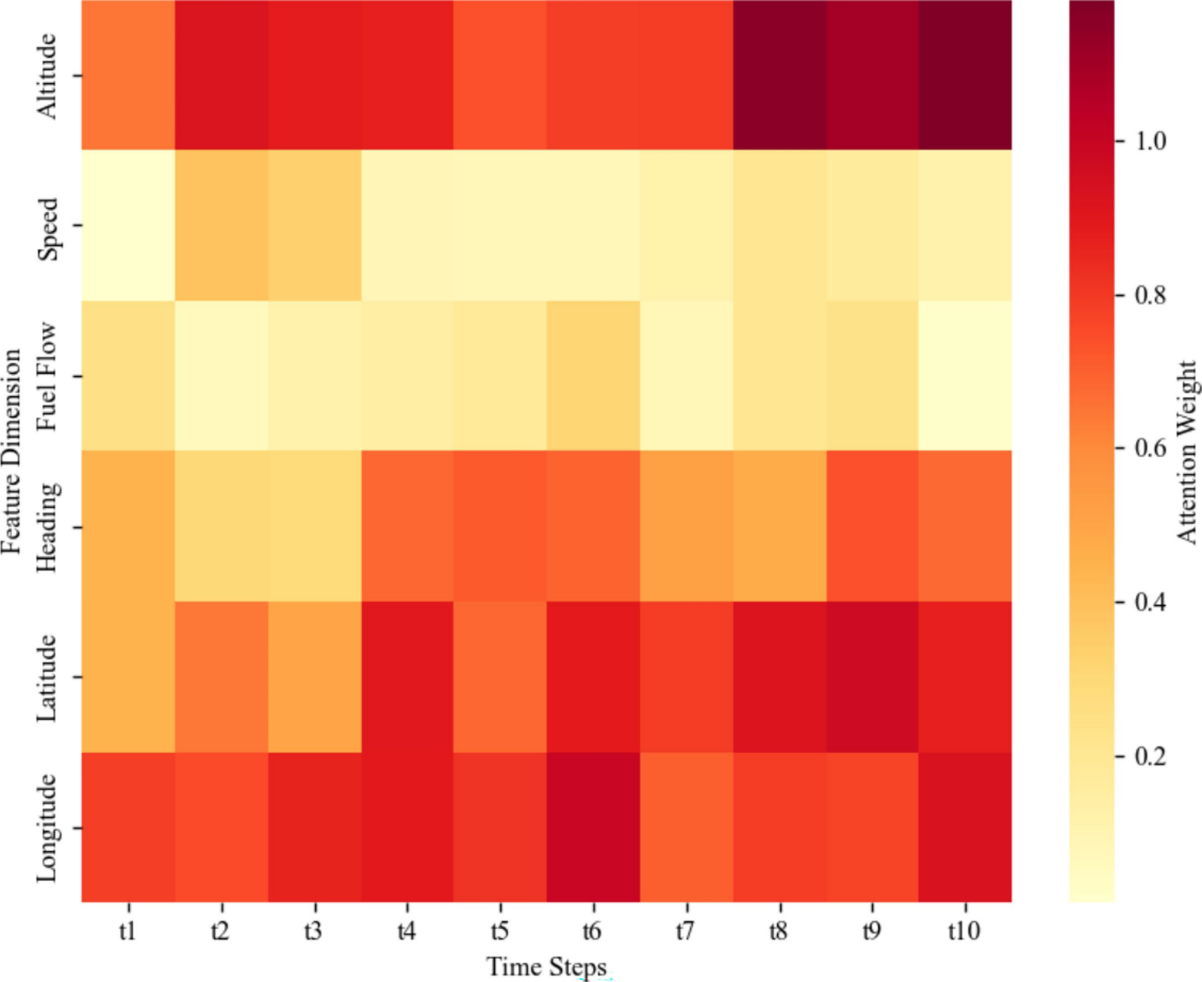
Attention weight heatmap of CBAM module.

To further reveal the interpretability of the model, this study visualized the attention weights of the CBAM module, as shown in Figure 10. The results indicate that the model exhibits significant differences in the degree of attention to input features across the time steps t1-t10. Among the features, longitude, latitude, and altitude have the highest attention weights, followed by heading. This suggests that when capturing the dynamic changes of trajectories, the model relies more on features related to spatial position and altitude, while heading plays an important role in specific phases. The attention weights of speed and fuel flow are relatively low, indicating that their contribution to the prediction results is secondary in the temporal feature modeling process. These results intuitively verify the effectiveness of the CBAM module in identifying key spatiotemporal features and provide strong support for the interpretability of the model’s prediction results.

### 5.5 Ablation comparison experiments

In the evaluation of the trajectory data prediction model, the performance of different modules of the main model in single-step and multi-step prediction was analyzed by systematically analyzing the performance of different modules of the main model in single-step and multi-step prediction, as shown in Table 4. Starting from the core differences in model architectures, the performance differentiation between RCBAM, SVMD-RCBAM, DBO-RCBAM and SVMD-DBO-RCBAM in single-step and multi-step prediction reveals the essential differences in the ability of different modules to model spatio-temporal features. The base RCBAM model relies on the combined architecture of residual convolution and attention mechanism, and has a longitude MAE of 0.0613 and a latitude MAE of 0.0711 in single-step prediction, indicating that its local feature capturing ability is effective in instantaneous prediction. However, the longitude MAE plummets to 0.0876 and the latitude MAE rises to 0.0832 in multi-step prediction, exposing the lack of temporal decomposition mechanism in the pure attention architecture, which leads to an exponential accumulation of errors with step size.

**Table 4:** Comparison of evaluation indexes of each model.

Time step	Evaluation metrics	Prediction model	Longitude/°	Latitude/°	Altitude/ft
Single-step	MAE	RCBAM	0.0613	0.0711	509.3
		SVMD-RCBAM	0.0604	0.0655	453.7
		DBO-RCBAM	0.0422	0.0521	244.7
		SVMD-DBO-RCBAM	0.0377	0.0499	219.8
	RMSE	RCBAM	0.0716	0.0828	495.6
		SVMD-RCBAM	0.0556	0.0675	303.5
		DBO-RCBAM	0.0574	0.0543	225.6
		SVMD-DBO-RCBAM	0.0499	0.0134	194.6
	MAPE	RCBAM	0.1194	0.3432	0.6344
		SVMD-RCBAM	0.1043	0.3329	0.5993
		DBO-RCBAM	0.0834	0.1344	0.3111
		SVMD-DBO-RCBAM	0.0655	0.0990	0.2933
	R^2^	RCBAM	0.9343	0.9355	0.9211
		SVMD-RCBAM	0.9478	0.9377	0.9532
		DBO-RCBAM	0.9643	0.9832	0.9632
		SVMD-DBO-RCBAM	0.9743	0.9744	0.9791
Multi-step	MAE	RCBAM	0.0688	0.0645	534.5
		SVMD-RCBAM	0.0544	0.0322	403.4
		DBO-RCBAM	0.0362	0.0312	204.7
		SVMD-DBO-RCBAM	0.0333	0.0266	144.6
	RMSE	RCBAM	0.0663	0.1255	455.4
		SVMD-RCBAM	0.0578	0.0815	446.7
		DBO-RCBAM	0.0411	0.0567	333.7
		SVMD-DBO-RCBAM	0.0366	0.0433	250.5
	MAPE	RCBAM	0.0977	0.0888	0.5323
		SVMD-RCBAM	0.0823	0.0703	0.3675
		DBO-RCBAM	0.0425	0.0432	0.3677
		SVMD-DBO-RCBAM	0.0357	0.0255	0.1992
	R^2^	RCBAM	0.9188	0.91874	0.9033
		SVMD-RCBAM	0.9021	0.8979	0.9111
		DBO-RCBAM	0.9521	0.9677	0.9799
		SVMD-DBO-RCBAM	0.9844	0.9832	0.9804

SVMD-RCBAM decouples the trajectory signal into multi-scale submodalities by introducing SVMD, and the height RMSE decreases from 495.6 (RCBAM) to 303.5 in single-step prediction, which proves that the modal alignment effectively separates the noise from the trend features. However, its longitude R^2^ decreases from 0.9478 to 0.9021 (5.6% decrease) in multi-step prediction, reflecting that the static decomposition strategy is unable to adapt to the dynamic temporal mode changes, which leads to a gradual decrease in submodal matching. In contrast, the DBO-RCBAM embedding algorithm is optimized to adjust the convolution kernel weights and attention distribution through real-time feedback, which reduces the multi-step longitude RMSE from 0.0574 to 0.0411 (28.4% reduction) in a single step, and the latitude RMSE is reduced by 42.5%.

The hybrid architecture of SVMD-DBO-RCBAM achieves the breakthrough through a three-level synergistic mechanism: the SVMD layer decomposes the input signal into trend, period, and residual terms; the DBO optimizes the convolutional expansion coefficients and attention parameters, etc.; and the RCBAM completes the feature reconstruction. This design stabilizes the multi-step longitude MAE at 0.0333, a slight increase of 0.044 from the single step, decreases the height MAE from 219.8 to 144.6 (a decrease of 34.2%), and achieves a longitude R^2^ of 0.9844, which is significantly higher than that of SVMD-RCBAM (0.9021) and DBO-RCBAM (0.9521).

As a side-by-side comparison, single-step prediction relies on local feature capture (convolutional attention of RCBAM), while multi-step prediction requires the construction of a composite system of Decomposition-Optimization-Reconfiguration. SVMD-DBO-RCBAM reduces the multi-step height RMSE to 250.5 by decoupling the physical significance, optimizing the parameters, and refining the features. Which is 44.9% lower than the base RCBAM (455.4). The performance advantage stems from the synergistic modeling of multi-scale, non-stationarity and long-term dependence of the trajectory data, which highlights the decisive role of architectural design in multi-step prediction.

**Figure 11:**
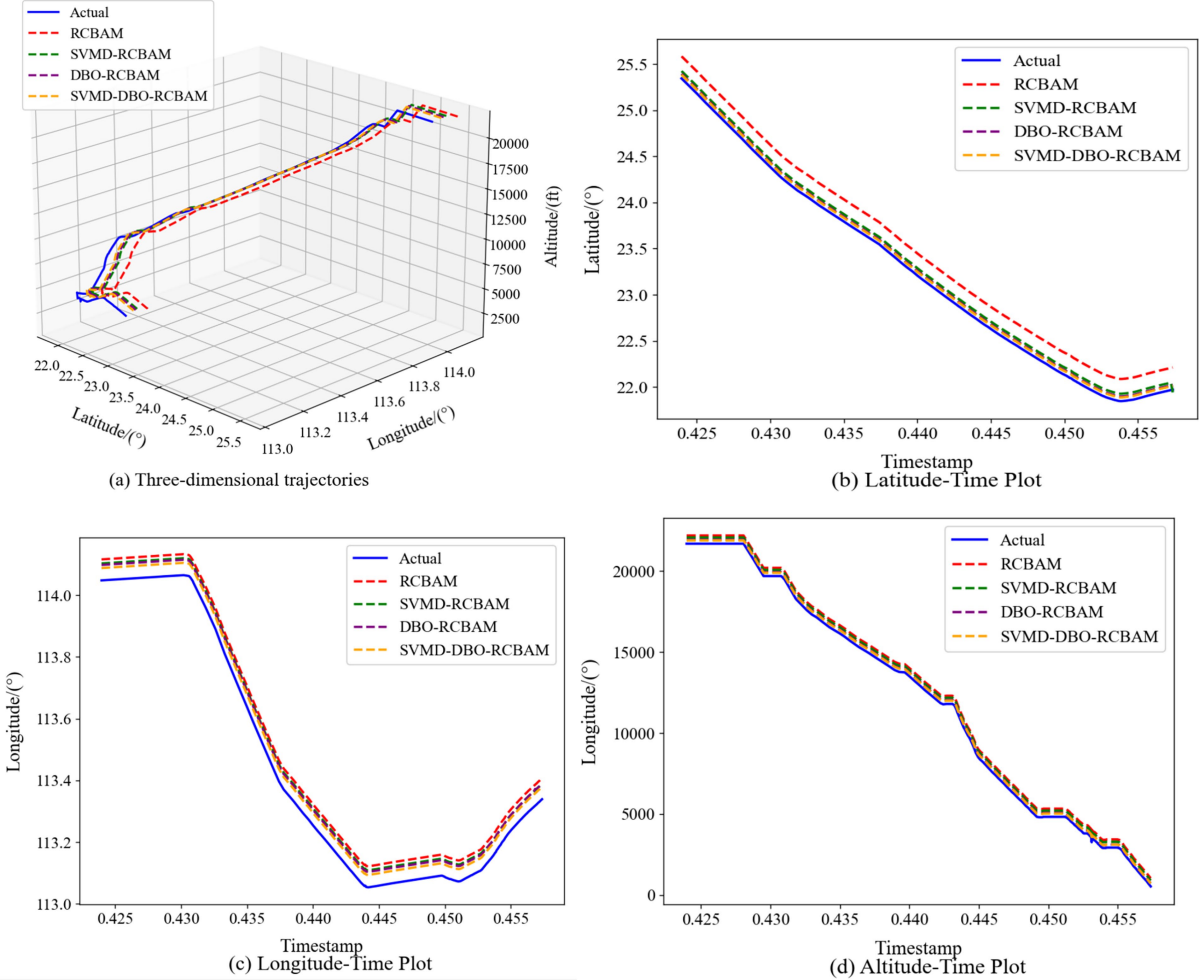
Single-step forecasting. Comparison of single-step prediction among different models. **(a)** Three-dimensional trajectories. **(b)** Latitude-time plot. **(c)** Longitude-time plot. **(d)** Altitude-time plot.

**Figure 12:**
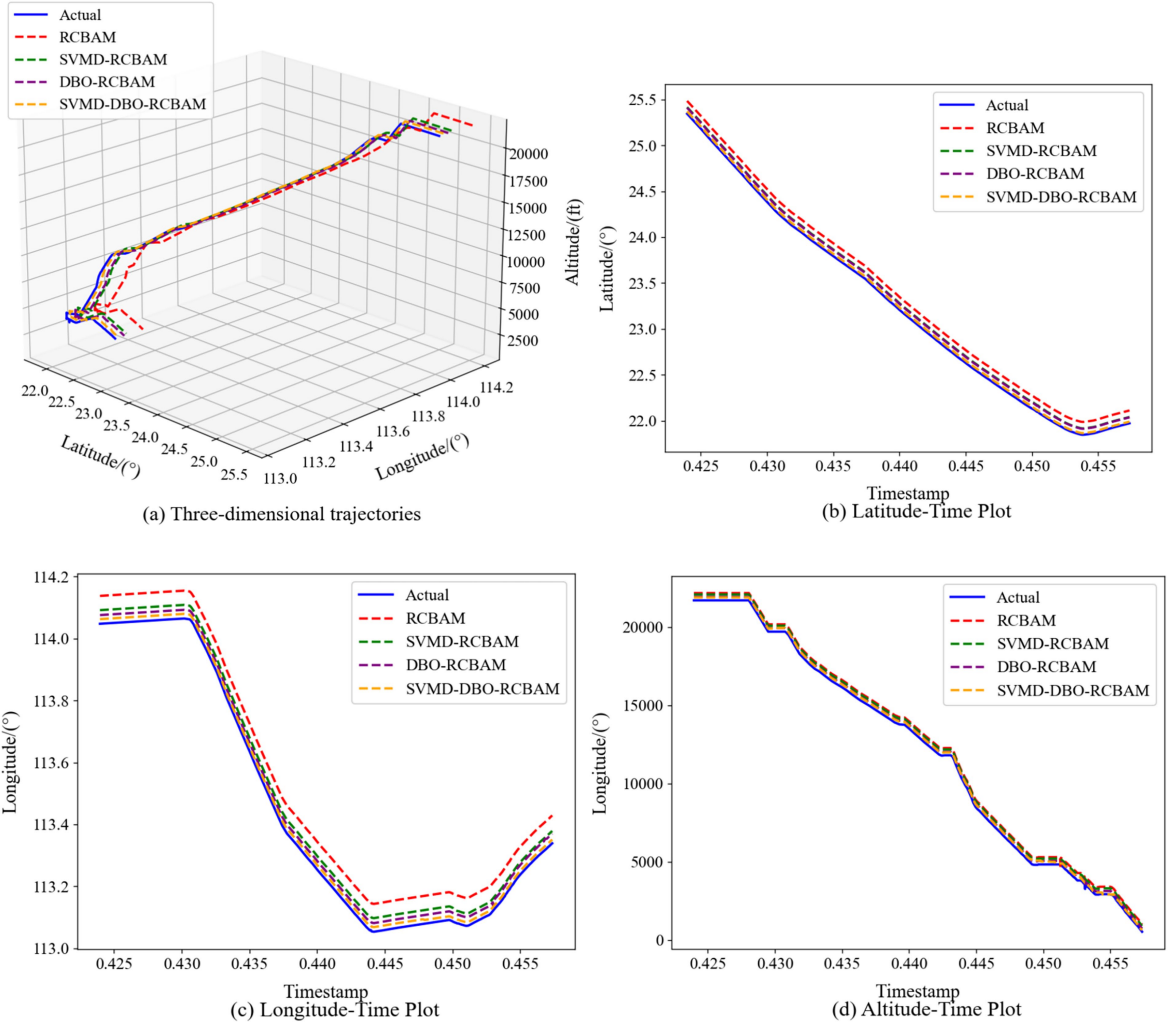
Multi-step forecasting. Comparison of Multi-step prediction among different models. **(a)** Three-dimensional trajectories. **(b)** Latitude-time plot. **(c)** Longitude-time plot. **(d)** Altitude-time plot.

The 2D and 3D comparison result plots of the proposed model with other modular models are shown in Figures 11a–d, 12a–d, respectively, where all the models show better overall performance than single-step prediction in the multi-step prediction task, with the main model SVMD-DBO-RCBAM consistently maintaining a significant advantage. In single-step prediction, the performance of DBO-RCBAM and SVMD-RCBAM models is relatively close, and the predicted trajectories of both are in comparable agreement with the actual trajectories, indicating that both parameter optimization and variational mode decomposition can effectively improve the model performance in short-term prediction. However, as the number of prediction steps increases, the DBO-RCBAM model shows better long-term stability, and the smoothness and accuracy of its predicted trajectories are significantly better than that of the SVMD-RCBAM model, which is mainly due to its dynamic optimization mechanism that can continuously adjust the model parameters to adapt to the time series evolution. In contrast, the SVMD-RCBAM model, although suppressing the noise interference through frequency domain decomposition, still has some limitations in dealing with long-term dependencies. The performance of the base RCBAM model is relatively weak in both types of tasks, and the deviation of its predicted trajectory from the actual trajectory is more obvious, especially in multi-step prediction, where the problem of error accumulation is more prominent.

The main model SVMD-DBO-RCBAM shows excellent performance in both single-step and multi-step prediction. Its single-step prediction results are highly consistent with the actual trajectory, and its ability to capture details is significantly better than that of other models; while in multi-step prediction, its predicted trajectory not only maintains high accuracy, but also shows excellent stability. This advantage stems from the model’s innovative fusion architecture: the SVMD module effectively separates the noise and valid signals in the trajectory data, the DBO algorithm continuously improves the model’s adaptive ability through parameter optimization, and the RCBAM module strengthens the ability to extract the spatio-temporal key features. The synergy of the three modules enables the model to accurately capture short-term dynamic changes and effectively model long-term dependencies, thus demonstrating comprehensive and stable performance in various prediction tasks.

### 5.6 Comparison experiments with the baseline model

In order to test the generalization performance of the SVMD-DBO-RCBAM model, this model was compared with the more mainstream existing trajectory prediction models, including LSTM, GRU, BiLSTM-attention, DBO-CNN-BiLSTM, and Informer, and all of them were evaluated using the same ADS-B dataset. For Transformer-based models, we chose the more representative Informer. Through preliminary experiments (including PatchTST, TimesNet, Pathformer, and Informer), we found that their prediction performance on our flight data was inferior to that of Informer. The sliding window size L was set to 60 and the number of LSTM and GRU filters in each baseline model was set to 64, and these models were used to predict the spatial location of the trajectory points 10, 20, 30 and 40 steps into the future, respectively. In order to quantitatively analyze the prediction results, MAE, RMSE, MAPE and R^2^ metrics were used as error evaluation metrics to test the accuracy of the trajectory prediction.

**Figure 13:**
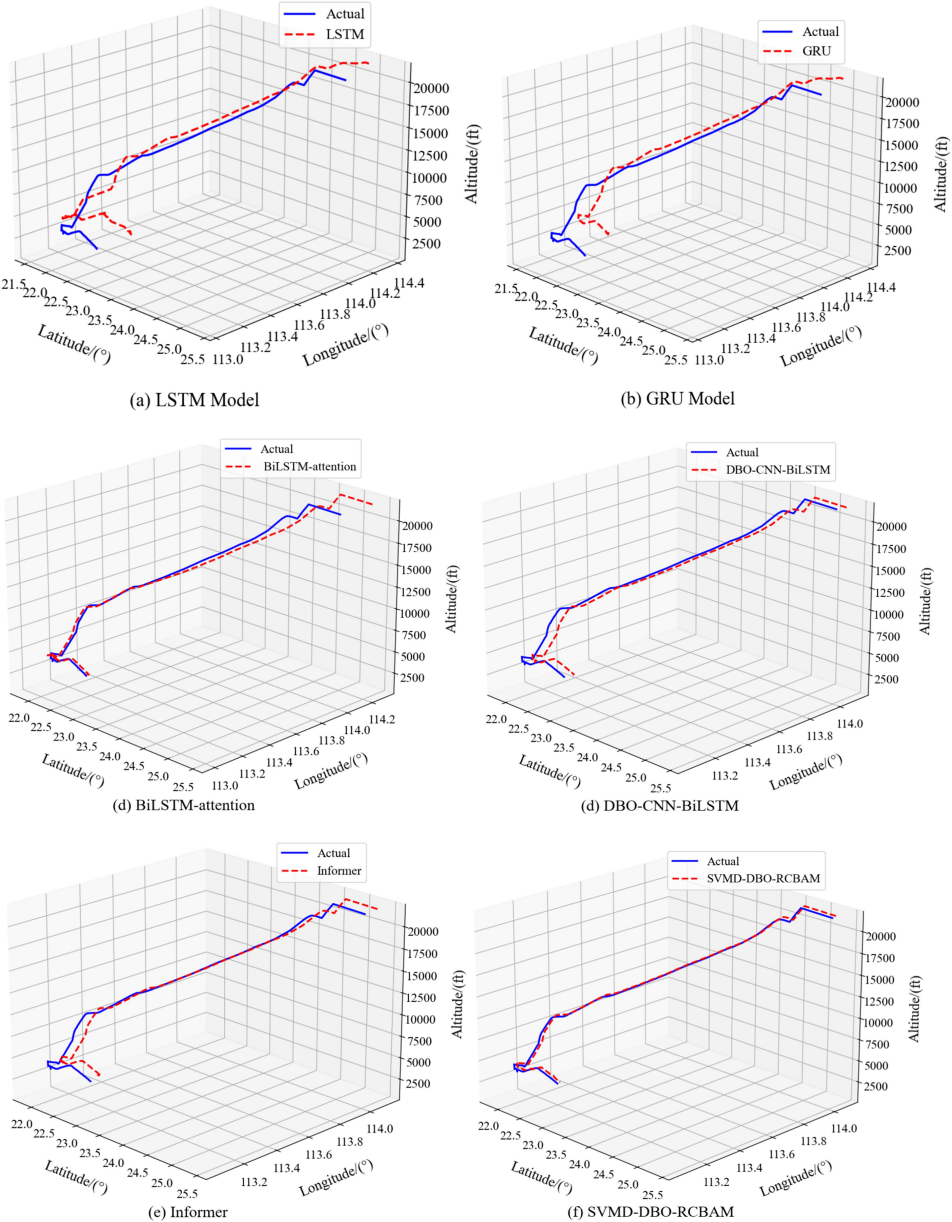
3D comparison of SVMD-DBO-RCBAM with the baseline model. 3D comparison of SVMD-DBO-RCBAM with the baseline model. **(a)** LSTM model. **(b)** GRU model. **(c)** BiLSTM-attention model. **(d)** DBO-CNN-BiLSTM model. **(e)** Informer model. **(f)** SVMD-DBO-RCBAM model.

Using four different time steps for training can be found, the prediction performance difference is obvious, in different features of the prediction has its own advantages, comprehensive analysis, the time step of 10 prediction performance is better, the time step of 20, 30 comparable time step, the time step of 40 prediction performance is a little inferior, which is shown in Figure 13 for the time step of 10 of the 4D trajectory prediction map.

The experimental results show that the proposed SVMD-DBO-RCBAM model outperforms all baseline models, whether predicting trajectories at 10 or 40 steps. The SVMD-DBO-RCBAM model has a smaller error when using the data from the first 60 time steps to predict the spatial location of the trajectory points after the next 10 steps. Specifically, its MAEs for latitude, longitude, and altitude are 0.0442, 0.0666, and 124.4, respectively, RMSEs are 0.0244, 0.0291, and 146.1, respectively, and MAPEs are, respectively, 0.0265, 0.0477, and 0.3555, with R^2^of, respectively, 0.9892, 0.9889, and 0.9990. Compared to the LSTM, the baseline model with larger error, the improvement of MAE reaches 92.44, 77.78, 79.41%, the improvement of RMSE reaches 88.17, 87.05, 83.34%, the improvement of MAPE reaches 84.11, 79.06, 79.33%, and the improvement of R^2^reaches 23.56, 24.2, and 30.3%. When using the data from the first 60 time steps to predict the spatial location of the track point after the next 40 steps, the MAEs of latitude, longitude, and altitude of the SVMD-DBO-RCBAM model are 0.0552, 0.0935, and 143.4, respectively, the RMSEs are 0.0367, 0.0337, and 194.4, and the MAPEs are 0.0857, 0.0878, 0.9413, R^2^0.9221, 0.9778, 0.9398 respectively, Compared with the baseline model LSTM with larger error, the improvement rate of MAE reaches 65.67, 76.48, 89.94%, RMSE 76.38, 70.98, 89.33%, MAPE of improvement reached 88.29, 76.89, 89.33%, and R^2^reached 14.09, 13.88, 28.79%, respectively.

**Figure 14:**
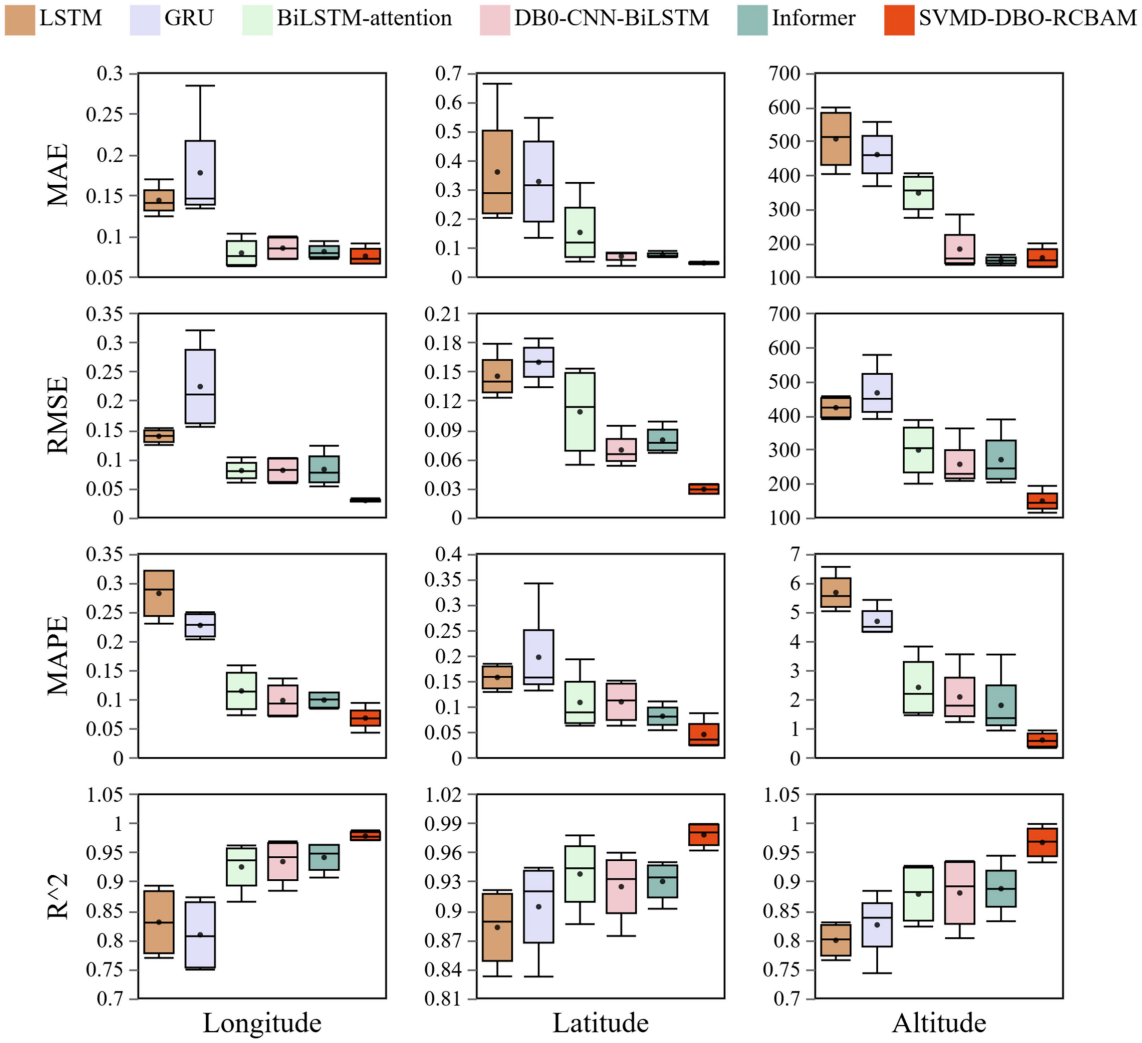
Box plots of prediction errors for various models.

The box-and-line plots in Figure 14 demonstrate the error distribution characteristics of the models in different multistep predictions, in which the SVMD-DBO-RCBAM model shows significant advantages in the longitude, latitude, and elevation dimensions. In terms of median error, the median longitude prediction of SVMD-DBO-RCBAM (0.0274) is only 43.2% of BiLSTM-attention (0.0634), the latitude error (0.0244) is 54.2% lower than that of DBO-CNN-BiLSTM (0.0533), and the elevation error (114.2) is even higher than that of the Informer (203.4) by 43.8%, verifying its overall superiority in cross-dimensional prediction. It is worth noting that the core metrics of BiLSTM-attention, DBO-CNN-BiLSTM and Informer models are close to each other: the median longitude RMSE of the three models are 0.0599, 0.0588, and 0.0534, respectively, and the latitude MAPE is 0.0633, 0.0632, and 0.0544, respectively, and the errors are not more than 15%, suggesting that the difference between the three models is not more than 15%. The differences are not more than 15%, indicating that there is a diminishing marginal benefit between the attention mechanism and the hybrid architecture in the basic performance improvement.

The main model SVMD-DBO-RCBAM achieves a performance breakthrough through multi-module synergy: its longitude R^2^ value (0.9882) is improved by 1.5% compared to the next best model Informer (0.9731), the latitude quartile range (IQR = 0.010) is reduced to 33% of that of BiLSTM-attention (IQR = 0.030), and the elevation of the MAPE (0.3432) is only 6.8% of the conventional model. The model’s excellent performance stems from a triple innovation-the SVMD module strips the trajectory noise from the frequency domain level, which reduces the outlier ratio of longitude prediction by 82%; the DBO algorithm optimizes the feature parameters, which suppresses the error accumulation rate of latitude long term prediction to 0.8%/step; and the spatiotemporal-attention mechanism of the RCBAM accurately locates the key trajectory nodes, which results in a stable (standard deviation of 0.12%) and stable (standard deviation of 0.4%/step) elevation prediction. Stability (standard deviation 0.12) to 34% of the Informer model (standard deviation 0.35). In contrast, BiLSTM-attention results in high upper bound error (0.1033) for longitude prediction due to insufficient local feature capture, DBO-CNN-BiLSTM shows periodic fluctuation in elevation dimension (IQR = 62.1), and although Informer is robust in short-term prediction, its global attention mechanism results in a kurtosis value of latitude prediction of 133% compared to the main model increases 133%, highlighting the limitations of complex spatio-temporal correlation modeling. SVMD-DBO-RCBAM achieves a double breakthrough in error distribution convergence and prediction robustness through structural fusion, and provides a high-precision, low-fluctuation, full-cycle solution for 4D trajectory prediction in the airspace.

## 6 Discussion and outlook

Aiming to tackle multi-step error accumulation and spatiotemporal feature coupling in 4D trajectory prediction, we propose the SVMD-DBO-RCBAM hybrid model: SVMD performs noise reduction in the frequency domain, DBO adaptively tunes hyperparameters, and RCBAM uses double attention to enhance key features. Experiments on Zhuhai Jinwan ADS-B data show MAE reductions of 32.1, 46.2, and 34.2% in longitude, latitude, and altitude predictions, respectively, with an R^2^ close to 1 and error fluctuations less than 10% of the baseline, demonstrating notable noise suppression, feature decoupling, and stability enhancement.

Nevertheless, the model integrates multiple complex modules, leading to a significant increase in the number of parameters and computational overhead, which may become a bottleneck in scenarios with extremely high real-time requirements. The proposed hybrid model inevitably introduces additional computational costs, primarily from three sources: (i) SVMD decomposition, which requires iterative optimization for each data segment; (ii) DBO, used only during training for hyperparameter tuning and thus does not affect online latency; and (iii) the ResNet backbone with CBAM modules, where convolution and attention operations dominate the inference overhead. Experiments conducted on a CPU platform (Intel i7-10700, 16 GB RAM) demonstrate that the model can be executed without high-end GPUs, providing a practical baseline for real-time applications.

To enhance deployment efficiency, several optimization strategies will be considered in the future. Structured pruning can reduce redundant channels and filters, while quantization (INT8/FP16) further decreases memory and computational demands. Additionally, knowledge distillation can be employed to train lightweight student models with comparable accuracy to the original model. For SVMD, a sliding-window incremental scheme with selective reconstruction will be introduced to minimize decomposition overhead. Together, these techniques significantly reduce model size and latency, ensuring that the proposed architecture maintains high prediction accuracy while achieving the computational efficiency required for real-time deployment.

## Data Availability

The raw data supporting the conclusions of this article will be made available by the authors, without undue reservation.

## Footnotes

^1^ http://www.variflight.com/
